# Characterization of a spray-dried candidate HPV L2-VLP vaccine stored for multiple years at room temperature

**DOI:** 10.1016/j.pvr.2017.03.004

**Published:** 2017-04-08

**Authors:** Julianne Peabody, Pavan Muttil, Bryce Chackerian, Ebenezer Tumban

**Affiliations:** a Department of Molecular Genetics and Microbiology, University of New Mexico School of Medicine, Albuquerque, NM 87131, USA; b Department of Pharmaceutical Sciences, College of Pharmacy, University of New Mexico, Albuquerque, NM 87131, USA; c Department of Biological Sciences, Michigan Technological University, Houghton, MI 49931, USA

**Keywords:** HPV vaccine, Bacteriophage L2-VLPs, Longevity, Formulation, Spray-drying, Thermostability

## Abstract

HPV infections are associated with human cancers. Although three prophylactic vaccines have been approved to protect against HPV infections, the vaccines require cold-chain storage and may not be suitable for third world countries with less developed refrigeration facilities. We previously developed a bacteriophage L2 virus-like particle (VLP)-based candidate vaccine, which elicited broadly protective antibodies against diverse HPV types. Spray-drying of MS2-16L2 VLPs into a dry powder enhanced the stability of these VLPs. Building on these studies, we assessed the long-term stability and immunogenicity of the spray-dried VLPs. Mice immunized with a single dose of spray-dried MS2-16L2 VLPs, which had been stored for 14 months at room temperature (RT), were partially protected from challenge with a high dose of HPV16, one year after immunization. However, immunization with two doses of MS2-16L2 VLPs stored at RT for 34 months elicited high titer anti-HPV antibodies. More importantly, this group of mice showed significant protection from HPV16, 4 months after immunization. These results suggest that spray-dried MS2-16L2 VLPs retain their effectiveness after long-term storage at RT, and may be suitable in third world countries with less developed refrigeration facilities.

## 1 Introduction

Infection with human papillomaviruses (HPVs) is associated with ~99.7% of cervical cancer, 46.9–71% of penile cancers/lesions, 71–90% of anal cancers, and 10–70% of oral, oropharyngeal, and laryngeal cancers [1], [2]. Fortunately, three prophylactic vaccines (Quadrivalent Gardasil, Cervarix, and Gardasil-9) have been approved to protect against HPV infections. Quadrivalent Gardasil (qGardasil) is approved for both girls and boys and protects against HPV16 and HPV18, which cause ~70% of cervical cancer cases and 53–79% of HPV-associated penile cancers. In addition to this, qGardasil protects against HPV6 and HPV11, which cause ~90% of genital warts. Cervarix protects against HPV16 and HPV18. Gardasil-9 protects against seven HPVs (HPV16, 18, 31, 33, 45, 52, and 58), which cause ~90% of cervical cancer cases, 86% of HPV-associated penile cancers, and 90–95% of HPV-associated anal cancers. Gardasil-9 also protects against HPV6 and HPV11 [1].

Although these vaccines are very effective in protecting against HPV infections, they all require continuous refrigeration to maintain their efficacy. This requirement comes with an additional challenge – the expense associated with refrigeration and the concern that the vaccines may be accidentally exposed to either freezing or warm conditions during storage or transportation, which may affect their efficacy. In fact, approximately 14–35% of all refrigerated vaccines (in general) are accidentally exposed to freezing temperatures during cold-chain storage/transportation [3]. HPV vaccines are formulated in alum adjuvant and exposure of alum-adjuvanted vaccines to freeze/thaw conditions may precipitate alum in the vaccines, thus reducing the immunogenicity of the vaccines. Precipitation of alum has been observed with alum-adjuvanted hepatitis B virus (HBV) and diphtheria-pertussis-tetanus vaccines [4], [5]. In a study with HBV vaccine, only 70% of immunized individuals in a rural Mongolia population developed a protective hepatitis B virus antibody response [4], [6]. Thus, the current alum-formulated HPV vaccines face freeze-thaw challenges.

Warm temperatures can also cause problems. Exposure of the vaccines to >25 °C (room temperature) for more than 72 h may affect their efficacy [7]. Unfortunately, developing (third world) countries may not have temperature-monitoring refrigeration facilities for vaccine storage and transportation. Even developed countries with adequate refrigeration facilities may sometimes face broken vaccine cold-chain systems. For example, Hurricanes Katrina and Matthew brought down power lines and damaged power poles [8], [9]. Power losses such as these can lead to elevated temperatures in refrigerators and consequently, loss in vaccine efficacy. With all of the above taken into consideration, the transportation & storage of cold-chain vaccines is critical and challenging; therefore, there is a need to improve the thermostability of HPV vaccines.

We had previously reported the development of bacteriophage MS2 virus-like particles (VLPs), displaying epitope 17–31 from the minor capsid protein (L2) of HPV16, as a candidate HPV vaccine [10]. In mouse model of HPV infection, MS2-16L2 VLPs elicited cross-protective antibody responses against diverse HPV types. For example, protection against HPV31 and HPV45 challenge was dramatically better than in mice immunized with qGardasil [11]. We also showed that the stability of these MS2-16L2 VLPs could be enhanced by spray-drying the VLPs into a dry powder. Spray-dried MS2-16L2 VLPs stored at room temperature or at 37 °C for 7–14 months were still immunogenic following immunization of mice [11], [12]. In this study, we assessed the immunogenicity and protection efficacy of the spray-dried MS2-16L2 VLPs following storage at room temperature for 34 months. We also assessed the longevity of protection in mice: i) one year after immunization with spray-dried MS2-16L2 VLPs stored at room temperature or at 37 °C for 14 months; ii) two years after immunization with lipopolysaccharide (LPS)-free MS2-16L2 VLPs.

## 2 Materials and methods

### 2.1 Expression of MS2-16L2 VLPs

MS2-16L2 VLPs were made by transforming *E. coli* C41cells with pDSP62-16L2 plasmid. pDSP62-16L2 plasmid expresses the single-chain dimer of bacteriophage MS2 coat protein displaying L2 epitope 17–31 from HPV16 [10]. Recombinant MS2-16L2 VLPs were expressed and purified as previously described [13], [14].

### 2.2 Formulation of VLPs into dry powder, stability studies, and removal of LPS from VLPs

MS2-16L2 VLPs were formulated with a combination of excipients as follows: First, an excipient solution that consisted of 85.4% mannitol, 1.71% trehalose, 0.85% dextran, 7.85% L-leucine, and 4.27% inositol was prepared. VLPs were then diluted to 1.2 mg/ml using this excipient solution (at a final concentration of 2.05%) and spray-dried using a Buchi Mini Spray Dryer B-290 with a standard two-fluid nozzle as previously described [11], [12].

To address the effect of contaminating lipopolysaccharide (LPS) on the longevity of our VLPs, LPS was removed from VLPs using 1% (final concentration) Triton X-114 as previously described [15].

### 2.3 Stability, immunization, and protection studies

For stability studies, MS2-16L2 VLPs were stored in air-tight containers at room temperature for 14–34 months after which they were reconstituted in sterile phosphate buffered saline buffer. An aliquot of the MS2-16L2 VLPs were observed under an electron microscope. Five μg of the MS2-16L2 VLPs stored for 14 months were used to immunize mice once (without alum hydroxide) and 5 μg of VLPs stored for 34 months were mixed with alum hydroxide and were used to immunize mice twice with alum hydroxide. Another group of mice was immunized only once with 5 µg of LPS-free MS2-16L2 VLPs (not spray-dried) without alum hydroxide. Control mice were immunized with MS2 VLPs. All immunizations were done by intramuscular injection. Two weeks after the last immunization, sera were collected from mice immunized with VLPs stored for 34 months at room temperature and anti-HPV L2 IgG antibody titers were determined by peptide end-point dilution ELISA as previously published [14].

For protection studies, HPV pseudovirus 16 (representing HPV16), encapsidating a reporter plasmid (pClucf) encoding both luciferase and green fluorescence protein (GFP), were made, purified, and used to vaginally infect immunized mice as previously described [14].

## 3 Results

### 3.1 Spray-dried MS2-16L2 VLPs are stable at room temperature for 34 months and they are protective against HPV16 infection

**Fig. 1:**
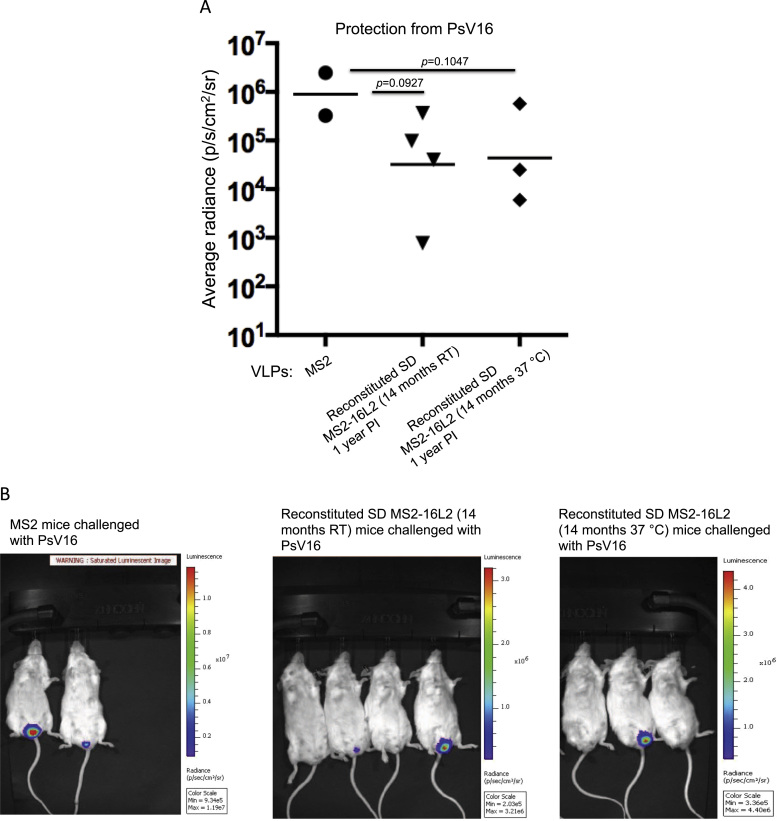
Protection from PsV16 infection, one year after immunization with reconstituted spray-dried (SD) VLPs. A) Mice were intramuscularly immunized once with 5 μg of spray-dried MS2-16L2 VLPs (stored at room temperature or 37 °C for 14 months) or control MS2 VLPs without alum hydroxide. One year after immunization, mice were vaginally infected with a high dose of HPV PsV16. Luciferase activity was measured forty-eight hours post challenge and average radiance (p/s/cm^2^/sr) values for each mouse was determined using Living Image 3.2 software. Each datum represents the average radiance of an individual mouse and each line represents the geometric mean radiance for each group. Statistical analysis was done using one-tailed unpaired *t*-test. B) IVIS images of bioluminescence signal in the genitals of mice. Average radiance values in A were determined from these images. Control MS2 mice (left image) compared to experimental mice showed high-saturated bioluminescence signal following infection with HPV PsV16.

We had previously devised a spray-dried candidate vaccine in which MS2-16L2 VLPs were formulated with a mixture of sugars (mannitol, trehalose, dextran and inositol) and an amino acid (L-leucine) into a dry powder. Spray-drying increased the long-term (7–14 months) stability of the VLPs at both room temperature and 37 °C without affecting its immunogenicity [11], [12]. Building on these studies, we assessed the longevity of immune protection, one year after immunization with the spray-dried VLPs that were stored at room temperature or 37 °C for 14 months. As shown in Fig. 1, mice immunized with a single dose (5 μg) of spray-dried MS2-16L2 VLPs without alum adjuvant were partially protected from vaginal infection with HPV pseudovirus (PsV) 16.

**Fig. 2:**
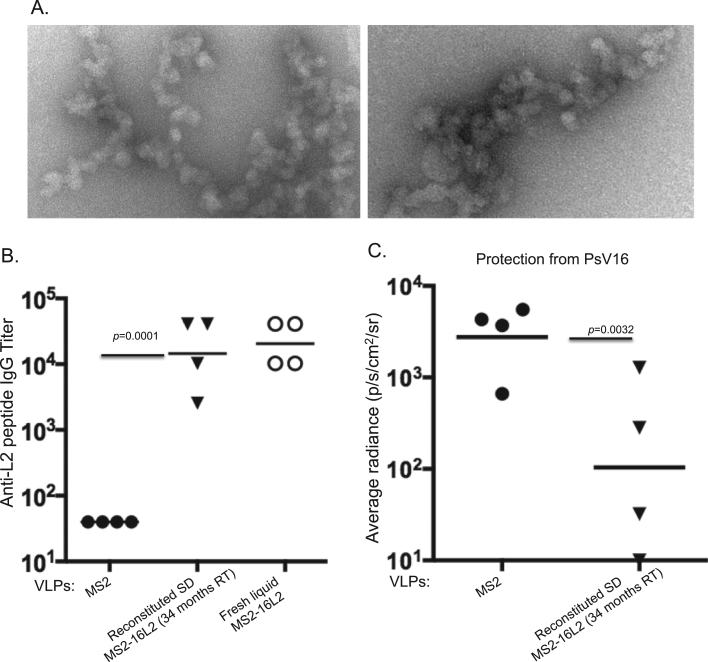
Thermostability, immunogenicity, and protective efficiency of spray-dried MS2-16L2 VLPs after storage for 34 months at room temperature. A) Spray-dried VLPs were reconstituted in sterile phosphate buffered saline solution and the VLPs were observed under an electron microscope at 70,000x. B) Balb/c mice were immunized twice with 5 µg of MS2-16L2 VLPs (from 2 A), fresh liquid MS2-16L2 VLPs or control MS2 VLPs (with alum hydroxide). Sera were collected two weeks after the last immunization and anti-L2 IgG antibody titers were determined by ELISA using HPV 16L2 (amino acid 17–31) as a target peptide. Statistical analysis was done using two-tailed unpaired *t*-test. C) Immunized mice in B were infected with 6.4×10^6^ PsV infectious units of HPV16, 4 months after immunizations. Luciferase activity, average radiance, and statistical analysis were determined as described in Fig. 1 legend.

Given the fact that spray-dried MS2-16L2 VLPs were stable and immunogenic after storage for up to 14 months at room temperature [12], we next assessed whether spray-dried MS2-16L2 VLPs were stable when stored at room temperature for up to 34 months and whether they could still elicit protective immune responses after long-term storage. Electron microscopy images of reconstituted spray-dried MS2-16L2 VLPs were inconclusive; they showed particulate structures that appeared to resemble aggregated VLPs (Fig. 2A). Nevertheless, mice immunized with two (5 μg) doses of the reconstituted spray-dried MS2-16L2 VLPs elicited high-titer antibodies (10^4^) against HPV L2 peptide representing epitope 17–31 from HPV16; antibody titers were similar to those from mice immunized with fresh liquid MS2-16L2 VLPs (Fig. 2B). To assess if these antibody titers – in reconstituted spray-dried MS2-16L2 VLPs-immunized mice – translate to immune protection, we vaginally infected the mice with HPV PsV16. As shown in Fig. 2C, a significant level of protection (p=0.0032) was observed in the mice immunized with the reconstituted spray-dried MS2-16L2 VLPs compared to those immunized with control MS2 VLPs. Thus, MS2-16L2 VLPs stored for nearly three years still elicited protective antibody responses.

### 3.2 LPS-free MS2-16L2 VLPs are protective two years after immunization

**Fig. 3:**
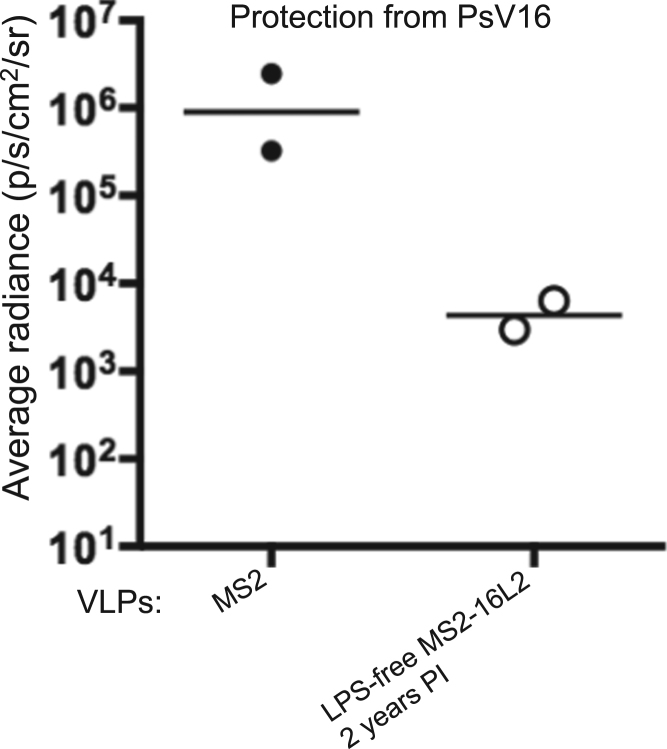
Longevity of protection in mice immunized with lipopolysaccharide (LPS)-free MS2-16L2 VLPs. Lipopolysaccharide was removed from VLPs using 1X Triton X-114. 5 µg LPS-free VLPs without alum hydroxide were used to immunize mice once. The mice were then vaginally infected with HPV PsV16, 2 years after immunization. Luciferase activity and average radiance were determined as described in Fig. 1 legend.

Because our MS2-16L2 VLPs are expressed and purified from bacteria, they contain contaminating LPS that may make it unsafe for human use. In addition, there was concern that contaminating LPS in our VLPs may be contributing to the immunogenicity of our MS2-16L2 VLPs; LPS is known to potentiate immune responses through its interaction with toll-like receptor 4. To address the latter, we removed LPS from the VLPs and assessed the longevity of protection two years following a single immunization with 5 µg of LPS-free MS2-16L2 VLPs (without alum hydroxide). Mice immunized with the LPS-free MS2-16L2 VLPs showed protection from vaginal infection with HPV PsV16 compared to control mice (Fig. 3).

## 4 Discussion and conclusion

Current HPV vaccines are predicted to offer ~70–90% protection against cervical cancer and up to 86% protection against HPV-associated penile cancer. Nevertheless, the vaccines require continuous cold-chain storage and may not be suitable for third world countries with less-developed refrigeration facilities or in rural villages with no refrigeration facilities at all. Additionally, some vaccines in rural areas are ignorantly transported using frozen ice packs directly from freezers, which accidentally expose vaccines to freezing conditions, precipitating alum in the vaccines, thus reducing the efficacy of vaccines [4], [5], [6]. With the aforementioned setbacks with cold-chain vaccines, we decided to formulate our candidate HPV vaccine, MS2-16L2 VLPs, into a thermostable product that would be potentially suitable for third world countries, especially rural areas that may lack refrigeration facilities. Spray-drying of MS2-16L2 VLPs into a dry powder increased the thermostability – without compromising immunogenicity – of the spray-dried VLPs when stored at room temperature or 37 °C for 14 months [12] compared to non-spray-dried MS2-16L2 VLPs, which disintegrated after one month of storage at room temperature [11]. As a follow-up to the spray-dried study, we showed that mice immunized with one dose (5 μg) of spray-dried MS2-16L2 VLPs are partially protected from vaginal infection with HPV PsV16, one year after immunization. One possible explanation for this partial protection is that mice were challenged with a high dose. As shown in Fig. 1B, the bioluminescence signal for MS2 control mice infected with PsV16 is saturated, which signifies high infectivity. A saturated bioluminescence signal implies the signal is above the limit of detection (a limitation of the imaging system) and as such, the signal is not accurately quantified by the IVIS Lumina II software. As such, the average radiances (p/s/cm^2^/sr), computed by the software for control mice were less than the actual values. At a lower challenge dose, protection may have been significant. Unfortunately, the mice could only be challenged once with the same type of HPV PsV; there is little or no infectivity – due to immunity from prior challenge – if the same mice are re-challenged with the same PsV type.

The level of protection can be enhanced further by immunizing with two doses of the spray-dried MS2-16L2 VLPs in alum hydroxide adjuvant. This view is supported by the fact that mice immunized with two doses of the spray-dried VLPs – stored at room temperature for 34 months – had similar antibody titers to mice immunized with two doses of fresh liquid MS2-16L2 VLPs (Fig. 2B). Moreover, mice immunized with the spray-dried VLPs – stored at room temperature for 34 months – were significantly protected from HPV PsV16, 4 months after immunization, even though the immunogen had aggregated VLPs (Fig. 2C). These results suggest that the efficacy of spray-dried VLPs stored at room temperature for more than 2 years may be enhanced if the doses of the reconstituted VLPs are increased and if the vaccine is delivered with alum hydroxide.

Successful removal of LPS from our MS2-16L2 VLPs [15] made our VLPs a candidate HPV vaccine that could potentially be evaluated for clinical use. In this study, we showed that removing the LPS from the VLPs does not adversely affect the longevity of protection two years after immunization. Taken together, MS2-16L2 VLPs is a candidate HPV vaccine that could be formulated into a thermostable vaccine for use in third world countries – where the majority of HPV-associated cancers occur – without the need for refrigeration.

## Conflict of interest

E Tumban and B Chackerian are inventors of L2-VLP related patent applications licensed to Agilvax Biotech. Interactions with Agilvax are managed by the University of New Mexico in accordance with its conflict of interest policies. The authors have no other relevant affiliations or financial involvement with any organization or entity with a financial interest in or financial conflict with the subject matter or materials discussed in the manuscript apart from those disclosed.
